# Liver-Specific Nonviral Gene Delivery of Fibroblast Growth Factor 21 Protein Expression in Mice Regulates Body Mass and White/Brown Fat Respiration

**DOI:** 10.1124/jpet.121.000514

**Published:** 2021-08

**Authors:** Nathaniel G. Girer, Victoria G. Rontoyanni, Aditya Joshi, Igor Patrikeev, Andrew J. Murton, Craig Porter, Massoud Motamedi, Cornelis J. Elferink

**Affiliations:** Department of Pharmacology and Toxicology (N.G.G., A.J., C.J.E.), Metabolism Unit, Department of Surgery (V.G.R., A.J.M., C.P.), Department of Ophthalmology and Visual Sciences, Center for Biomedical Engineering (I.P., M.M.), Sealy Center of Aging (V.G.R., A.J.M.), The University of Texas Medical Branch at Galveston, Galveston, Texas; Department of Pharmacology and Toxicology, College of Pharmacy, University of Oklahoma Health Sciences Center, Oklahoma City, Oklahoma (A.J.); and Division of Developmental Nutrition, Department of Pediatrics, University of Arkansas for Medical Sciences, Little Rock, Arkansas (C.P.)

## Abstract

Viral-mediated in vivo gene delivery methods currently dominate among therapeutic strategies within the clinical and experimental settings, albeit with well documented limitations arising from immunologic constraints. In this study, we demonstrate the utility of nonviral hepatotropic in vivo gene delivery of unpackaged expression constructs, including one encoding fibroblast growth factor 21 (FGF21). FGF21 is an important hepatokine whose expression positively correlates with therapeutic outcomes across various animal models of obesity. Our data demonstrate that FGF21 expression can be restored into the livers of immunocompetent FGF21 knockout mice for at least 2 weeks after a single injection with an FGF21 expression plasmid. In wild-type C57BL6/J mice, in vivo transfection with an FGF21-expressing plasmid induced weight loss, decreased adiposity, and activated thermogenesis in white fat within 2 weeks. Furthermore, in vivo FGF21 gene delivery protected C57BL6/J mice against diet-induced obesity by decreasing adiposity and increasing uncoupling protein 1–dependent thermogenesis in brown fat and by boosting respiratory capacity in subcutaneous and perigonadal white fat. Together, the data illustrate a facile and effective methodology for delivering prolonged protein expression specifically to the liver. We contend that this method will find utility in basic science research as a practical means to enhance in vivo studies characterizing liver protein function. We further believe our data provide a rationale for further exploring the potential clinical utility of nonviral gene therapy in mouse models of disease.

**Significance Statement:**

This study presents a valuable method for nonviral gene delivery in mice that improves upon existing techniques. The data provide a rationale for further exploring the potential clinical utility of nonviral gene therapy in mouse models of disease and will likely enhance in vivo studies characterizing liver protein function.

## Introduction

Gene therapy as an approach to disease management/treatment has proven quite promising, and the United States has already approved multiple gene therapies for clinical use. One example is Zolgensma, a single-injection, expression vector–based gene therapy used to treat spinal muscular atrophy (Dangouloff and Servais, 2019). Gene therapy approaches are especially useful for the treatment of hepatic diseases, as previous work has demonstrated the liver can readily absorb DNA molecules delivered intravenously (Nicklin et al., 1998). To date, liver-specific gene delivery has successfully been achieved through either hydrodynamic delivery or adeno-associated virus (AAV) infection. However, hydrodynamic delivery is harsh and technically demanding, requiring very large intravenous injection volumes (∼8%–10% body mass) delivered in a matter of seconds. AAV gene therapy is meanwhile encumbered by the need to generate recombinant viruses as viable high-titer virus preparations. Moreover, the viruses elicit a robust innate immune response in immunocompetent organisms that precludes repeated administration of the viruses (Manno et al., 2006). These limitations not only place constraints on their clinical application but also undermine the utility of expression constructs in research animal models. Therefore, the development of more amenable strategies for gene delivery warrants investigation.

Fibroblast growth factor 21 (FGF21) is a unique member of the fibroblast growth factor family of proteins that plays a key role in regulating energy metabolism (BonDurant and Potthoff, 2018; Kliewer and Mangelsdorf, 2019). Circulating FGF21 primarily derives from the liver and acts upon brown and white adipose tissue to increase mitochondrial respiration (Fisher et al., 2012; Markan et al., 2014; Girer et al., 2019) via obligate binding to the fibroblast growth factor receptor 1c and *β*-Klotho proteins (Adams et al., 2012; Foltz et al., 2012). In brown adipose tissue (BAT), FGF21 increases the expression and activity of uncoupling protein 1 (UCP1), a thermogenic protein that generates heat through the uncoupling of ATP synthesis from respiration (Kwon et al., 2015; Porter, 2017). In white adipose tissues, FGF21 increases mitochondrial respiration through the AMP-activated protein kinase-Sirtuin-1-Peroxisome proliferator-activated receptor-gamma coactivator- 1alpha pathway (Chau et al., 2010). Circulating FGF21 can additionally act upon the brain to promote BAT thermogenesis and the browning of white adipose tissue through increased adrenergic signaling (Douris et al., 2015).

Numerous physiologic conditions have been shown to induce FGF21 synthesis, including prolonged fasting, supraphysiological glucose concentrations, Endoplasmic Reticulum stress, and cold exposure (Lundåsen et al., 2007; Iizuka et al., 2009; Fisher et al., 2012; Jiang et al., 2014). Notably, the administration of recombinant FGF21 protein in various animal models of obesity consistently achieves therapeutic outcomes including weight loss, improved glucose tolerance/insulin resistance, increased energy expenditure, and reduced adiposity (Coskun et al., 2008; Xu et al., 2009; Kwon et al., 2015). Given the remarkable success FGF21 has demonstrated in treating obesity in animals, researchers are actively investigating FGF21 for its potential use in a clinical setting. Although recombinant wild-type FGF21 has performed poorly in clinical trials to date, other FGF21 analogs with greater thermostability have shown improved efficacy (Adams et al., 2013; Gaich et al., 2013). FGF21 mimetics, such as the monoclonal antibody mimAb1, are also being investigated as an alternative approach to activating the antiobesity properties of FGF21 in target tissues (Foltz et al., 2012).

In this study, we demonstrate the efficacy of nonviral in vivo gene delivery to transfect liver cells with different protein expression constructs in mice. Given the liver-specific origin of circulating endogenous FGF21 and its ability to facilitate weight loss and reduced adiposity when used in various models of obesity, we envisioned utilizing this nonviral gene therapy strategy to deliver Hemagglutinin (HA)-tagged FGF21 to the liver in vivo. We show that FGF21 gene delivery in wild-type C57BL6/J mice increased hepatic FGF21 production and drove physiologic changes including weight loss, decreased adiposity, and increased respiration within adipose tissue. We also demonstrate that nonviral FGF21 gene delivery protected against diet-induced obesity in mice. Our strategy importantly improves upon previous attempts at FGF21 gene delivery via hydrodynamic delivery that required large intravenous injection volumes unsuitable for the clinical setting (Gao et al., 2014) and demonstrates that gene delivery via intravenous administration of unpackaged plasmid DNA is a viable methodology for promoting long-term, hepatotropic protein expression in an experimental setting.

## Materials and Methods

### Mice

All experiments were performed with female C57BL6/J mice obtained from Jackson Laboratories (Bar Harbor, ME), unless otherwise stated. FGF21 LKO (hepatocyte-specific FGF21 conditional knockout) mice were generated via crossing *Fgf21^fl/fl^* [strain designation B6.129S6(SJL)-Fgf21^tm1.2Djm^/J] mice to B6.Alb-Cre^ERT2^ mice. B6.Alb-Cre^ERT2^ mice were obtained from Ben Strangers (University of Pennsylvania, Philadelphia, PA) with permission from Pierre Chambon (Pasteur Institute, Paris, France) (Feil et al., 1996). B6.Alb-Cre^ERT2^ mice express a tamoxifen-inducible Cre recombinase. To induce gene deletion, mice were administered a single dose of 75 mg/kg tamoxifen in corn oil on 3 consecutive days. All mice were housed on corn cob bedding in a pathogen-free, climate- and temperature-controlled facility and were given ad libitum access to standard rodent chow (Harland Tekland 7912; Madison, WI) or a 60% kcal very-high-fat diet (Research Diets D12492; New Brunswick, NJ) and water. Upon sacrifice, sections of liver were snap-frozen in liquid nitrogen and stored at −80°C. All mouse experiments were conducted humanely and in accordance with the Animal Care and Use Committee Guidelines at the University of Texas Medical Branch at Galveston (protocol 0109034E, approved September 2019) and Guide for the Care and Use of Laboratory Animals as adopted and promulgated by the US National Institutes of Health.

### Histologic Analyses

Upon sacrifice, sections of subcutaneous white adipose tissue, perigonadal white adipose tissue, and liver were fixed in 10% neutral buffered formalin (StatLab, McKinney, TX) for 48 hours and then submitted to the Research Histopathology Core (University of Texas Medical Branch) for slide preparation and H&E staining. Adipocyte area measurements were performed using the automated open-source Adiposoft plug-in and ImageJ software (Galarraga et al., 2012; Schneider et al., 2012). Measurements were constrained to adipocytes with diameters 25–125 pixels in experiments with standard chow–fed mice and 30–300 pixels for experiments with high-fat diet–fed mice.

### Plasmids

All plasmid constructs employed for this study were generated on the commercially available pLIVE background (Mirus Bio, Madison, WI). pLIVE-SEAP was purchased directly from Mirus Bio. pLIVE-*rluc* was generated via subcloning red luciferase cDNA from pLenti-UBC-redLuc vector kindly provided by Dr. Jeffery Fair (University of Texas Medical Branch at Galveston, Galveston, TX). pLIVE-FGF21-HA was generated by subcloning the full-length FGF21 open reading frame encoded with an in-frame C-terminal HA tag from pCMV3-FGF21-C-HA (catalog number MG50421-CY; Sino Biologic, Wayne, PA) into pLIVE-Empty. pLIVE-Empty (Mirus Bio, Madison, WI) was used as a negative control.

### Luciferase Reporter

For in vivo luciferase imaging, we employed the IVIS Spectrum in vivo imaging system (Perkin Elmer, Waltham, MA). The Promega Luciferase assay system (Promega, Madison, WI) was used to quantify luciferase activity as per the manufacturer’s instructions. Briefly, liver homogenates were prepared using passive lysis buffer and then plated onto a black 96-well plate containing 100 μl of luciferase assay reagent. Luciferase activity was then measured on the Glomax Explorer Multimode microplate reader (Promega, Madison, WI).

### In Vivo Delivery of Plasmid

We employed the commercially available In vivo-JetPEI-Gal (Polyplus Transfection, New York, NY) system as per the manufacturer’s instructions to deliver plasmid to the liver in vivo. Briefly, 40 µg of endotoxin-free plasmid in sterile PBS and 6.4 µl of transfection reagent were diluted separately in 10% glucose, incubated at room temperature for 15 minutes, and then combined. For each mouse, 200 μl of the final mixture was injected into the tail vein.

### SEAP Detection

Blood was collected either via tail vein bleed or cardiac puncture at sacrifice. Whole blood was incubated at room temperature for 30 minutes and then centrifuged for 10 minutes at 5000*g* to collect the serum. Then, 1 μl of serum was used to measure SEAP activity using a commercially available kit (Phospha-Light SEAP Assay Kit; Applied Biosystems).

### RNA/Protein Analyses

RNA was isolated from tissues using Trizol Reagent as per the manufacturer’s instructions (Life Technologies, Carlsbad, CA). cDNA was prepared as previously described (Harper et al., 2013). Polymerase chain reaction was carried out using Taq polymerase as per the manufacturer’s instructions and the following primers: *Fgf21*, forward: 5′-GGGGATTCAACACAGGAGAA-3′, reverse: 5′-AGGGCCTCAGGATCAAAGTGA-3′; and *Glyceraldehyde 3-phosphate dehydrogenase*, forward: 5′-ACGGCAAATTCAACGGCACAGTCA-3′, reverse: 5′-CATTGGGGGTAGGAACACGGAAGG-3′. Protein analyses were conducted as previously described (Girer et al., 2019) using the following antibodies: anti-HA C29F4 (Cell Signaling Technologies, Danvers, MA), anti-FGF21 Y-16 (clone sc-81946; Santa Cruz Biotechnology, Dallas, TX), or anti-actin (clone 13E5; Cell Signaling Technologies, Danvers, MA). Quantification of protein expression, normalized to actin, was performed using ImageJ software (Schneider et al., 2012).

### ELISA

Primary antibody against FGF21 (clone sc-81946; Santa Cruz Biotechnology, Dallas, TX) was diluted 1:200 into 100 μl total volume of coating buffer (Na_2_CO_3_/NaHCO_3_, pH 9.6), applied to an opaque 96-well plate, and incubated overnight at 4°C. Wells were then washed (wash buffer: 150 mM NaCl, 0.05% Tween-20) and incubated at 37°C for 3 hours with 100 μl sterile PBS + 5% dry milk. Wells were subsequently washed and then incubated for 1 hour at 37°C with 100 μl of serum diluted 1:200 into sterile PBS. Wells were washed again and then incubated with 100 μl of horseradish peroxidase–conjugated secondary antibody against HA (clone C29F4; Cell Signaling Technologies, Danvers, MA) diluted 1:6000 in sterile PBS at 37°C for 1 hour. Wells were washed again, and 60 μl of QuantaRed horseradish peroxidase substrate (Millipore-Sigma, Darmstadt, Germany) was prepared as per the manufacturer’s instructions and then applied. Fluorescence was measured after a 5-minute incubation at room temperature using wavelengths 530–570 nm for excitation and 585–630 for detection. All samples were corrected for background fluorescence using PBS as the negative control.

### High-Resolution Mitochondrial Respiration Assay

Mitochondrial respiration was measured as previously described (Porter et al., 2016; Girer et al., 2019; Bhattarai et al., 2020). Briefly, white or brown adipose tissue was immediately collected upon sacrifice and then submerged in ice-cold preservation BIOPS buffer (2.77 mM CaK_2_EGTA, 7.23 mM K_2_EGTA, 20 mM imidazole, 20 mM taurine, 50 mM 2-(N-morpholino)ethanesulfonic acid hydrate, 0.5 mM Dithiothreitol, 6.56 mM MgCl_2_·6H_2_O, 5.77 mM Na_2_ATP, and 15 mM Na_2_Phosphocreatine; pH 7.1). Respiration was then measured within 10 hours of collection using an Oxygraph-2k respirometer.

### Statistical Analyses

All data points in this manuscript represent individual mice that have been pooled from two or more planned experiments to achieve a minimum of *n* = 5. ELISA assays were performed in technical triplicates. Statistical analyses were performed as described in the figure legends using GraphPad Prism Software version 5.0.

## Results

### In Vivo Gene Delivery Using Unpackaged Plasmid Is a Suitable Technique for Introducing Long-Term, Liver-Specific Protein Expression

**Fig. 1.**
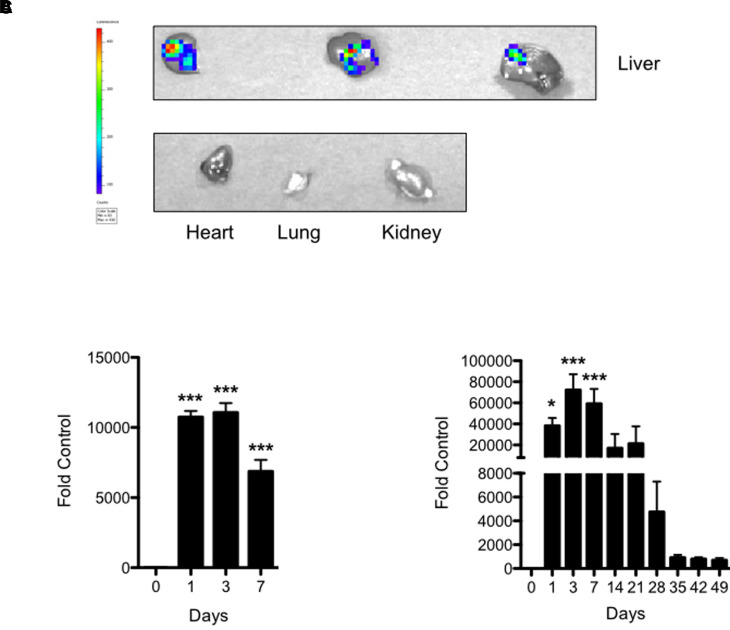
Successful, liver-specific in vivo delivery of long-term protein expression. (A) In vivo delivery of pLIVE-*rluc* vector appears to occur exclusively in the liver, as no reporter activity was detected in other extrahepatic tissues. (B) Luciferase activity in the liver persists around 7500-fold of control at 7 days postinjection. (C) In vivo delivery of pLIVE-SEAP in C57BL6/J mice results in potent serum SEAP activity for up to 3 weeks and is still detected at 1000-fold of control at 7 weeks postinjection. Images shown are representative of three animals. Numerical data are presented as means ± S.E.M. (*n* = 3 per time point). Statistically significant differences versus control (time point zero), as determined by one-way ANOVA with Dunnett post-test or repeated measures ANOVA, are shown as *P < 0.05 or ***P < 0.001.

Countless studies have demonstrated the ability to deliver unpackaged small interfering RNA and other short-length oligonucleotides in vivo. We first explored whether we could successfully, and specifically, target the liver with a naked, luciferase-expressing plasmid construct using a similar in vivo delivery strategy. To do so, we cloned red firefly luciferase cDNA into pLIVE vector (pLIVE-*rLuc*) and then delivered the vector into C57BL6/J mice via tail vein injection. Figure 1A demonstrates that luciferase activity is specific to the liver, as no luciferase activity was detected in the heart, lungs, or kidneys. Luciferase activity was increased to as much as 10,000 times that of pLIVE-Empty–transfected control animals and persisted at 6800-fold control at 7 days post-transfection (Fig. 1B). Having successfully demonstrated target organ specificity, we next determined the maximum length of time that the in vivo delivery technique can maintain protein expression. To do so, we employed pLIVE-SEAP, a commercially available control plasmid that encodes for serum embryonic alkaline phosphatase. Because SEAP is secreted into the blood, it allows for recurring in vivo monitoring of hepatic protein expression in the same animal without sacrifice. Figure 1C shows that after a single injection of vector, serum SEAP activity peaked at approximately 72,000-fold of control 3 days postinjection and remained readily detectable at 700-fold above the control signal after 7 weeks. Together, the data in Fig. 1 demonstrate that our in vivo transfection protocol using unpackaged plasmid constitutes a facile and effective strategy for delivering long-term, liver-specific protein expression in vivo.

### Reintroduction of FGF21 into FGF21 Conditional Knockout Mice

**Fig. 2.**
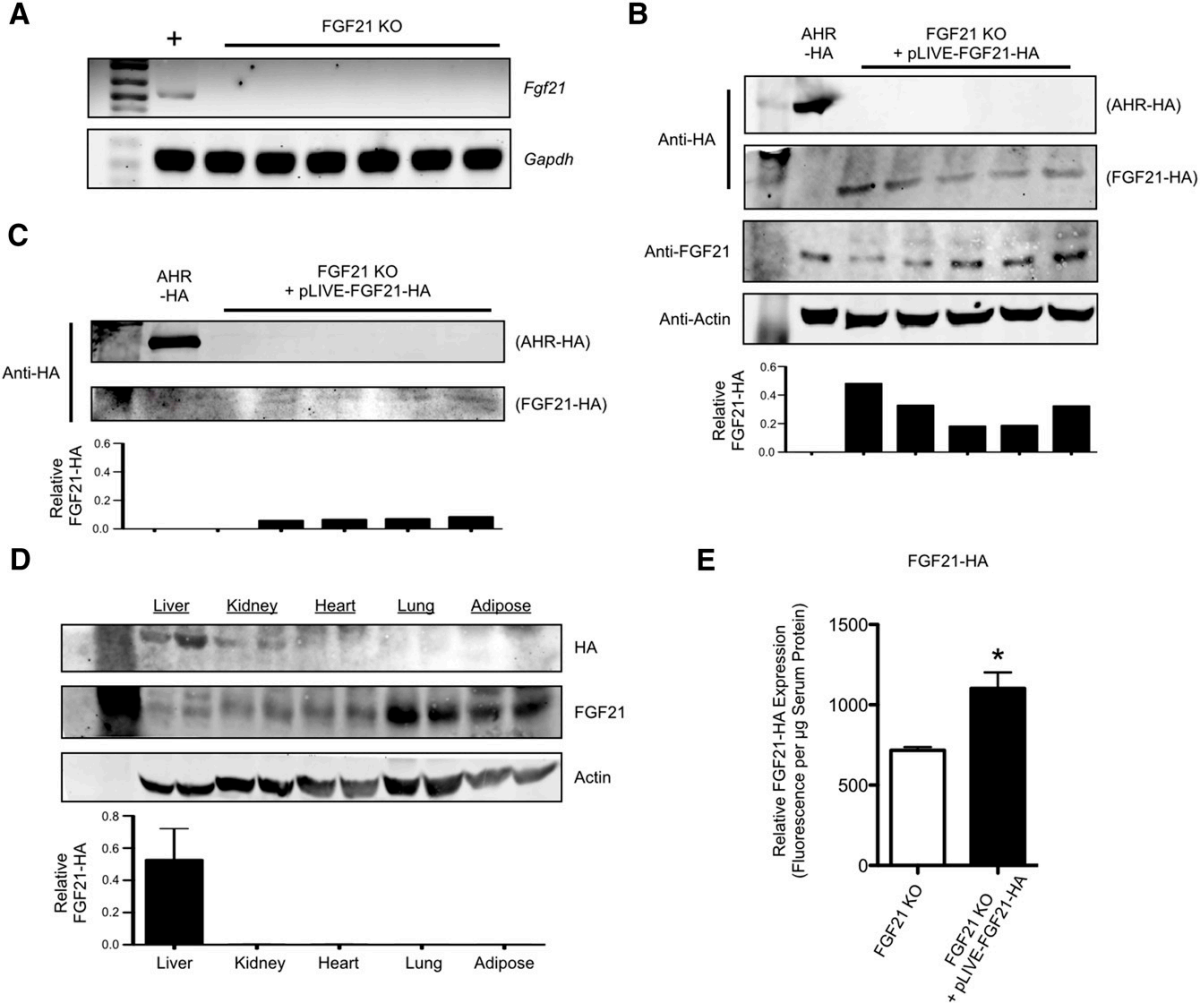
Reintroduction of FGF21 protein expression into FGF21 LKO (liver-specific conditional knockout) mice via delivery of an unpackaged plasmid. (A) Polymerase chain reaction analysis confirming successful loss of *Fgf21* transcript in nontransfected tamoxifen-treated FGF21 LKO mice. *Gapdh* (Glyceraldehyde 3-phosphate dehydrogenase) is used as a positive control. (B) In vivo transfection with pLIVE-FGF21-HA establishes FGF21-HA protein expression in FGF21 LKO mice. The lane containing in vitro–translated HA-tagged Aryl Hydrocarbon Receptor (AHR-HA) serves as a positive control for the specificity of the anti-HA antibody. (C) At 4 weeks postinjection, hepatic FGF21-HA expression is largely abated. (D) FGF21-HA expression is primarily restricted to the liver in transfected FGF21 LKO mice (*n* = 4 per tissue). (E) FGF21-HA is present in the serum of reconstituted mice (*n* = 5), indicating that vector-encoded protein maintains its physiologic characteristics. Unless otherwise stated, individual bars within densitometry graphs are representative of individual mice. Statistically significant differences, as determined by a Student’s *t* test, are denoted as *P < 0.05.

FGF21 is an important metabolic hormone whose expression can confer weight loss and improved metabolic function in various models of obesity (Kliewer and Mangelsdorf, 2019). The introduction of FGF21 expression into hepatocytes might accordingly be suitable as a therapeutic approach to alleviate the obese disease state. We therefore sought to demonstrate the feasibility of reintroducing FGF21 expression into a hepatocyte-targeted FGF21 conditional knockout mouse model (FGF21 LKO). Because FGF21 is a secreted protein (Markan et al., 2014), we used a C-terminal HA-tagged form of the protein to track its expression in serum and distinguish it from endogenous FGF21. Figure 2A confirms the successful deletion of hepatic FGF21 mRNA and protein in the livers of FGF21 LKO mice. In FGF21 LKO mice transfected with pLIVE-FGF21-HA, we achieved successful reintroduction of hepatic FGF21-HA protein expression and observed the continued expression of FGF21 at 2 weeks postinjection (Fig. 2B). The specificity of the HA antibody to HA, and not FGF21, was verified via employing in vitro–translated HA-tagged aryl hydrocarbon receptor protein as a simultaneous FGF21-negative/HA-positive control. Consistent with our previous experiment using pLIVE-SEAP–transfected mice (Fig. 1C), FGF21-HA expression is substantially reduced by 4 weeks postinjection (Fig. 2C). In subsequent experiments, we therefore examined the effects of FGF21 gene delivery at 2 weeks postinjection. To confirm the tissue specificity of our technique, we analyzed FGF21-HA expression in extrahepatic tissues at 2 weeks postinjection. The quantified data show substantial liver FGF21-HA protein expression, with negligible protein detected in the kidney, heart, lung, or perigonadal white adipose tissues (Fig. 2D). Given that FGF21 is a hepatokine, we next conducted an ELISA assay to measure serum HA-FGF21 concentrations to determine that the FGF21-HA is readily secreted into the serum after transfection. Indeed, our data confirm that FGF21-HA was readily detected in the serum at 2 weeks postinjection (Fig. 2E). Taken together, these data demonstrate the feasibility of nonviral in vivo FGF21 gene therapy as a tool for promoting prolonged hepatic FGF21 protein expression.

### In Vivo FGF21 Gene Delivery Produces Physiologic Changes in C57BL6/J Mice

**Fig. 3.**
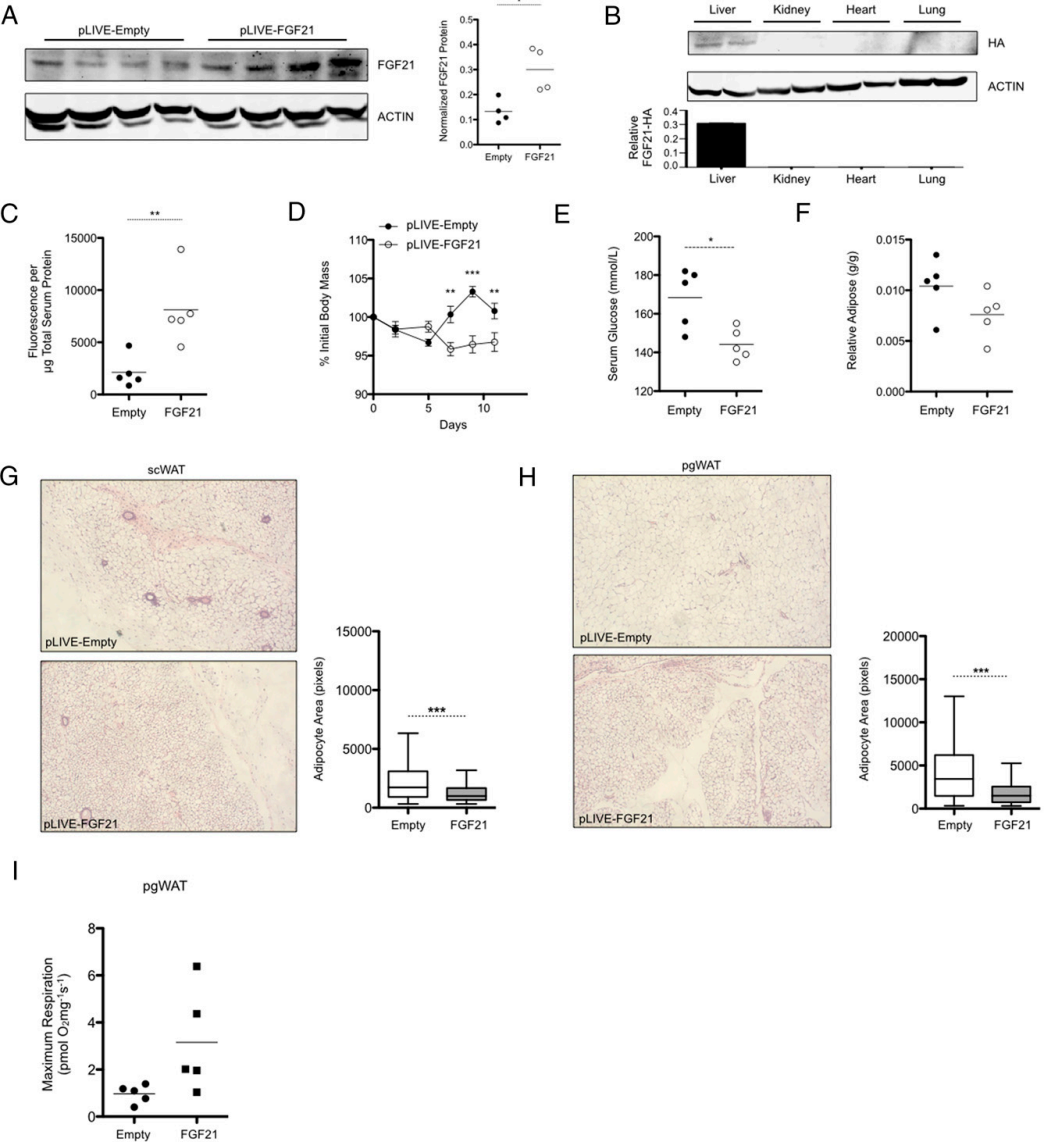
In vivo delivery of pLIVE-FGF21-HA increases hepatic FGF21 protein expression and produces physiologic changes in C57BL6/J mice. (A) FGF21 expression in transfected mice exceeds physiologic expression levels. (B) FGF21-HA expression is exclusively found in the livers of transfected mice (*n* = 4 per tissue). (C) Transfection with pLIVE-FGF21-HA results in readily detectable concentrations of HA-tagged protein in the serum at 2 weeks postinjection. (D) C57BL6/J mice transfected with pLIVE-FGF21-HA exhibit significantly reduced body mass gain relative to controls and (E) a significant reduction in fed-state serum glucose concentrations. (F) The relative quantity of pgWAT trends lower in pLIVE-FGF21-HA–transfected mice. (G) Mice transfected with pLIVE-FGF21-HA display reduced adiposity within scWAT as well as (H) pgWAT. (I) Maximal respiratory capacity of pgWAT trends higher after transfection with pLIVE-FGF21-HA. Microscopy images were obtained at 100× magnification and are representative of three individual mice. Unless otherwise stated, numerical data are presented as means ± S.E.M. (*n* = 5 per group), as a scattered dot plot with the mean denoted by a horizontal bar, or as a box-and-whiskers plot using the Tukey method. Statistical analyses were performed using a Student’s *t* test or two-way ANOVA with Bonferroni post-tests. Statistically significant differences are shown as *P < 0.05, **P < 0.01, or ***P < 0.001.

Having demonstrated the capacity to successfully reconstitute hepatic FGF21 expression in FGF21 LKO mice, we next sought to establish that hepatic FGF21-HA production in transfected wild-type mice will elicit the anticipated physiologic changes associated with elevated FGF21 levels. Figure 3A demonstrates that FGF21 protein levels in mice transfected with pLIVE-FGF21-HA are reproducibly and significantly increased 2.3-fold beyond the normal physiologic levels observed in mice transfected with pLIVE-Empty vector. FGF21-HA protein appeared to be limited exclusively to the liver, as no HA protein was detected in several extrahepatic tissues (Fig. 3B). As observed in reconstituted FGF21 LKO mice, injection with pLIVE-FGF21-HA produced a detectable increase in serum HA protein (Fig. 3C), suggesting that circulating FGF21-HA protein was established in these wild-type mice. In addition to increasing FGF21 protein levels beyond physiologic levels, transfection with pLIVE-FGF21-HA resulted in a decrease in body mass (slope = −0.3208 g/d, *P* = 0.00025 vs. pLIVE-Empty) for 2 weeks after injection (Fig. 3D), consistent with the known effects of FGF21 (Kliewer and Mangelsdorf, 2019). Serum glucose concentrations during the nonfasted state were also significantly reduced 14% in pLIVE-FGF21-HA–transfected wild-type mice relative to controls (Fig. 3E), in line with the known effects of exogenous FGF21 administration (Kwon et al., 2015). Although falling short of statistical significance (*P* = 0.11), relative perigonadal white adipose tissue (pgWAT) mass trended lower in mice transfected with pLIVE-FGF21-HA (Fig. 3F). We nevertheless observed a pronounced decrease in adipocyte size in histologic samples of subcutaneous white adipose tissue (scWAT) from FGF21-transfected mice, which corresponded with a significant 41% decrease in mean adipocyte area (Fig. 3G). We likewise observed a visible reduction in adipocyte size within pgWAT, analogous to a significant 8% decrease in mean adipocyte area (Fig. 3H). Consistent with these findings, thermogenic UCP1-dependent mitochondrial respiration in pgWAT trended upward but was short of statistical significance (*P* = 0.09) (Fig. 3I).

### In Vivo Gene Therapy Using pLIVE-FGF21-HA Successfully Reduces Obesity in Very-High-Fat Diet–Fed Mice

**Fig. 4.**
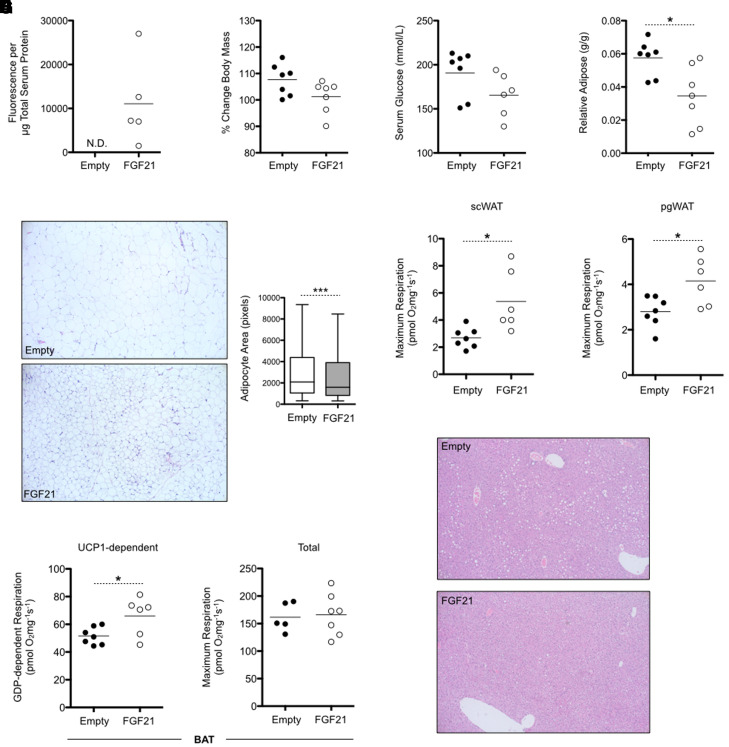
Nonviral in vivo FGF21 gene delivery protects against diet-induced obesity in C57BL6/J mice. (A) Circulating FGF21-HA is abundantly detected in the serum of pLIVE-FGF21-HA–transfected mice at 2 weeks postinjection. N.D., not detected. (B) Compared with pLIVE-Empty–transfected control mice, delivery of pLIVE-FGF21-HA to the liver produces a near-significant reduction of weight gain in response to high-fat diet (HFD) challenge after 2 weeks. (C) Fed-state serum glucose concentrations are modestly reduced in FGF21-transfected mice. (D) The relative mass of pgWAT is significantly lower in HFD-fed mice transfected with pLIVE-FGF21-HA. (E) Adipocytes are visibly and quantitatively smaller in scWAT collected from mice overexpressing FGF21 and (F) demonstrate a significantly greater maximal capacity for mitochondrial respiration. (G) Similarly, pgWAT adipocytes from pLIVE-FGF21-HA–transfected mice display a significant increase in the maximum mitochondrial respiratory capacity. (H) Thermogenic, UCP1-dependent respiration in BAT is significantly higher in FGF21-expressing mice, but maximum BAT respiratory capacity remains comparable to control mice. (I) Transfection with pLIVE-FGF21-HA protects against HFD-associated liver steatosis. Microscopy images shown are at 100× magnification and representative of three individual mice. Numerical data (*n* = 5 per group) are presented as a scattered dot plot with the mean denoted by a horizontal bar or as a box-and-whiskers plot using the Tukey method. Statistically significant differences, as determined by a Student’s *t* test, are shown as *P < 0.05 or ***P < 0.001.

To explore the potential of utilizing in vivo FGF21 gene therapy for the treatment of obesity, we subjected C57BL6/J mice to a very-high-fat diet for 10 weeks and then transfected the mice with either pLIVE-Empty control vector or pLIVE-FGF21-HA. Consistent with our previous observations, HA-tagged FGF21 was readily detected in the serum of pLIVE-FGF21-HA–transfected mice but was not detectable in pLIVE-Empty–transfected mice (Fig. 4A) at 2 weeks postinjection. Figure 4B demonstrates that elevated serum FGF21 levels in FGF21-transfected mice were associated with a modest 6% decrease in weight gain relative to control mice, falling just short of statistical significance (*P* = 0.069). Nonfasting concentrations of serum glucose trended 13% lower (*P* = 0.1031) in pLIVE-FGF21-HA–transfected mice (Fig. 4C). In contrast, transfection with pLIVE-FGF21-HA significantly reduced the ratio of pgWAT to body mass by 40% (Fig. 4D) and resulted in visibly smaller adipocytes within scWAT deposits that corresponded with a 39% decrease of mean adipocyte area (Fig. 4E). Figure 4F shows that maximal mitochondrial respiratory capacity was significantly increased 2-fold in scWAT collected from FGF21-transfected mice. Respiratory capacity was also significantly increased 1.5-fold in pgWAT from pLIVE-FGF21-HA–transfected mice relative to empty vector controls (Fig. 4G). In BAT, increased circulating FGF21 paralleled a significant 1.3-fold increase in GDP-dependent thermogenic respiration without any change in maximum respiratory capacity (Fig. 4H). In the liver, transfection with pLIVE-FGF21-HA vector resulted in visibly reduced lipid deposition. Together, these data demonstrate that our method of nonviral in vivo FGF21 gene therapy protects against the effects of diet-induced obesity.

## Discussion

As the primary site for detoxification and metabolism, the liver constantly imports and exports molecules from the bloodstream and is therefore an ideal target for in vivo delivery of DNA via intravenous injection. To date, liver-specific gene delivery is primarily achieved through either hydrodynamic delivery or AAV infection. The studies presented here demonstrate that the delivery of unpackaged expression constructs to hepatocytes in vivo can also be achieved using a more facile transfection strategy. Employing our technique, we provide evidence of the sustained expression of multiple different proteins in mouse liver (i.e., luciferase, SEAP, HA-FGF21). Our success in doing so suggests that this strategy is highly versatile and likely suitable for the expression of many liver proteins.

FGF21 delivery in mice was previously employed successfully via the use of hydrodynamic gene delivery (Gao et al., 2014). This methodology is technically challenging, however, and requires large injection volumes (≈8%–10% body mass) over a very short duration (≈5–10 seconds). This requirement places constraints on its utility in clinical applications, and hence, greater interest has been devoted into viral delivery systems. Our gene delivery method only utilizes a small 200-μl injection volume in mice that approximates to 0.6% body mass, making this method more tractable. Recently, Jimenez et al. (2018) successfully employed adenovirus-based FGF21 gene therapy in mouse models of obesity. Compared with our method of gene delivery, viral gene therapy requires the additional steps of generating recombinant viruses and high-titer viable virus preparations and, moreover, elicits a robust innate immune response in immunocompetent organisms (Manno et al., 2006). Although viral gene therapy can indeed produce long-lasting expression, this strategy is still inherently transient because efficient readministration with the same AAV serotype is hindered by anti-AAV neutralizing antibodies produced after the initial AAV treatment. In contrast, our delivery method produces robust FGF21 expression for up to 4 weeks. Moreover, based upon findings with the hydrodynamic gene delivery paradigm, transfections with nonpackaged DNA vectors exhibit markedly diminished immune responses that would render subsequent transfections feasible (Yokoo et al., 2016). We contend that the combination of a small injection volume, the potential to repeatedly administer expression constructs, and the facile nature of the method not dependent on generating viral particles render this strategy superior to other approaches described in the literature.

Consistent with the known actions of FGF21, our data show that increased circulating FGF21 concentrations in pLIVE-FGF21-HA–transfected mice maintained on standard rodent chow diminished weight gain and improved glucose uptake (Markan et al., 2014). In addition, increased serum FGF21-HA levels were associated with reduced adiposity, due in part to a concurrent increase in white fat respiration. FGF21 was previously shown to suppress weight gain in animal models of obesity (Adams et al., 2013; Gao et al., 2014; Jimenez et al., 2018). In mice challenged with a very-high-fat diet, transfection with pLIVE-FGF21-HA resulted in reduced weight gain and adiposity, in line with the antiobesity effects of FGF21 previously reported in the literature (Coskun et al., 2008). BAT thermogenic activity and white fat respiration were also significantly increased in FGF21-transfected mice, matching data from our previous study that demonstrate increased energy expenditure in animals with elevated circulating FGF21 levels (Girer et al., 2019). Our data corroborate both the efficacy of FGF21-based therapies in mouse models of obesity and the mechanisms through which these therapeutic effects occur.

In summary, our data present a novel methodology for the controlled delivery of hepatic FGF21 expression in mice and demonstrate the successful implementation of this strategy to reduce the effects of diet-induced obesity in female C57BL6/J mice. The data particularly underscore the utility of this gene delivery method in basic science research as a valuable means to enhance in vivo studies focused on liver protein function and provide a precedent for further exploring the clinical utility of nonviral gene therapy. Current research in the laboratory continues to successfully employ this technique to reconstitute the expression of other liver proteins in corresponding conditional gene knockout mouse models.

## Abbreviations

AAV: adeno-associated virus
BAT: brown adipose tissue
FGF21: fibroblast growth factor 21
pgWAT: perigonadal white adipose tissue
scWAT: subcutaneous white adipose tissue
UCP1: uncoupling protein 1
